# Common Genetic Variation in the Human *FNDC5* Locus, Encoding the Novel Muscle-Derived ‘Browning’ Factor Irisin, Determines Insulin Sensitivity

**DOI:** 10.1371/journal.pone.0061903

**Published:** 2013-04-25

**Authors:** Harald Staiger, Anja Böhm, Mika Scheler, Lucia Berti, Jürgen Machann, Fritz Schick, Fausto Machicao, Andreas Fritsche, Norbert Stefan, Cora Weigert, Anna Krook, Hans-Ulrich Häring, Martin Hrabě de Angelis

**Affiliations:** 1 Department of Internal Medicine, Division of Endocrinology, Diabetology, Angiology, Nephrology and Clinical Chemistry, Eberhard Karls University Tübingen, Tübingen, Germany; 2 Institute for Diabetes Research and Metabolic Diseases of the Helmholtz Centre Munich at the University of Tübingen, Tübingen, Germany; 3 German Centre for Diabetes Research (DZD), Neuherberg, Germany; 4 Institute of Experimental Genetics, Helmholtz Centre Munich, German Research Centre for Environmental Health, Neuherberg, Germany; 5 Department of Diagnostic and Interventional Radiology, Section on Experimental Radiology, Eberhard Karls University Tübingen, Tübingen, Germany; 6 Department of Internal Medicine, Division of Nutritional and Preventive Medicine, Eberhard Karls University Tübingen, Tübingen, Germany; 7 Department of Physiology and Pharmacology, Karolinska Institute, Stockholm, Sweden; 8 Chair for Experimental Genetics, Technical University Munich, Freising, Germany; Kunming Institute of Zoology, Chinese Academy of Sciences, China

## Abstract

**Aims/hypothesis:**

Recently, the novel myokine irisin was described to drive adipose tissue ‘browning’, to increase energy expenditure, and to improve obesity and insulin resistance in high fat-fed mice. Here, we assessed whether common single nucleotide polymorphisms (SNPs) in the *FNDC5* locus, encoding the irisin precursor, contribute to human prediabetic phenotypes (overweight, glucose intolerance, insulin resistance, impaired insulin release).

**Methods:**

A population of 1,976 individuals was characterized by oral glucose tolerance tests and genotyped for *FNDC5* tagging SNPs. Subgroups underwent hyperinsulinaemic-euglycaemic clamps, magnetic resonance imaging/spectroscopy, and intravenous glucose tolerance tests. From 37 young and 14 elderly participants recruited in two different centres, muscle biopsies were obtained for the preparation of human myotube cultures.

**Results:**

After appropriate adjustment and Bonferroni correction for the number of tested variants, SNPs rs16835198 and rs726344 were associated with *in vivo* measures of insulin sensitivity. Via interrogation of publicly available data from the Meta-Analyses of Glucose and Insulin-related traits Consortium, rs726344’s effect on insulin sensitivity was replicated. Moreover, novel data from human myotubes revealed a negative association between *FNDC5* expression and appropriately adjusted *in vivo* measures of insulin sensitivity in young donors. This finding was replicated in myotubes from elderly men.

**Conclusions/interpretation:**

This study provides evidence that the *FNDC5* gene, encoding the novel myokine irisin, determines insulin sensitivity in humans. Our gene expression data point to an unexpected insulin-desensitizing effect of irisin.

## Introduction

The importance of adipose tissue-derived hormones, collectively termed adipokines, for the regulation of glucose, lipid, and energy metabolism was convincingly shown, and it appears by now very plausible that dysregulated adipokine secretion significantly contributes to the pathogenesis of human metabolic diseases (i.e., obesity, atherosclerosis, type 2 diabetes) [1]. More recently, it was recognized that skeletal muscle and liver are also able to secrete, e.g., upon metabolic or physical stress, substantial amounts of metabolically active hormones, in analogy termed myokines and hepatokines, respectively [2]–[5]. Pathophysiological roles of individual myokines, such as interleukin-6 [6], and hepatokines, such as sex hormone-binding globulin and fetuin-A [7]–[9], in the development of human metabolic diseases are currently emerging.

A novel intriguing myokine, termed irisin, was very recently described by Boström et al. [10]. Irisin is released upon cleavage of the plasma membrane protein fibronectin type III domain-containing protein 5 (FNDC5). Expression of its gene was shown to be driven by muscle-specific transgenic overexpression of the exercise-responsive transcriptional co-activator peroxisome proliferator-activated receptor (PPAR)-γ co-activator-1α (PGC-1α) and, more physiologically, by three weeks of free wheel running in mice and by ten weeks of supervised endurance exercise training in humans [10]. After FNDC5 cleavage by a still unknown protease, irisin is released from muscle cells, enters the circulation, and is detectable in murine and human plasma [10]. Irisin treatment of differentiating primary murine preadipocytes induced, in a PPAR-α-dependent manner, the expression of brown fat genes (including *Ucp1*) [10], pointing to trans-determination and/or trans-differentiation of white adipose precursor cells [11]. This finding is in keeping with the observation of subcutaneous white adipose tissue ‘browning’ in PGC-1α-transgenic mice due to an increase in brown adipocyte number [10]. Finally, adenoviral *Fndc5* overexpression in mice increased energy expenditure (probably via enhanced thermogenesis) and improved obesity and insulin resistance induced by high-fat feeding [10].

Whether irisin or the *FNDC5* gene, encoding its membrane-resident protein precursor (MIM ID *611906), is involved in human metabolic disease is currently unknown. Therefore, we assessed in 1,976 German individuals at increased risk for type 2 diabetes whether common single nucleotide polymorphisms (SNPs; with minor allele frequencies [MAFs] ≥0.05) in the human *FNDC5* locus contribute to the prediabetic phenotypes overweight, glucose intolerance, insulin resistance, or impaired insulin release. In addition, we examined whether *in vitro FNDC5* gene expression in human myotubes reflects prediabetes-related metabolic *in vivo* traits of the donors.

## Materials and Methods

### 

#### Ethics statement

The study adhered to the Declaration of Helsinki, and its protocol was approved by the local ethics boards (Ethics Committees of the Eberhard Karls University Tübingen and the Karolinska Institute Stockholm). From all participants, informed written consent to the study was obtained.

#### Subjects

An overall study group of 1,976 White European individuals from Southern Germany was recruited from the ongoing Tübingen Family study for type 2 diabetes (TÜF) that currently encompasses more than 2,300 participants at increased risk for type 2 diabetes (i.e., non-diabetic individuals with family history of type 2 diabetes and/or diagnosis of impaired fasting glycaemia [12]. All subjects underwent the standard procedures of the protocol: assessment of medical history, smoking status, and alcohol consumption habits, physical examination, routine blood tests, and OGTTs. Selection of the overall study group was based on (i) the absence of newly diagnosed diabetes and (ii) the availability of complete phenotypic data sets. The participants were not taking any medication known to affect glucose tolerance, insulin sensitivity, or insulin secretion. From the overall study group, a subgroup of 486 subjects voluntarily agreed to undergo a hyperinsulinaemic-euglycaemic clamp procedure, a subgroup thereof (N = 360) additionally underwent MRI and magnetic resonance spectroscopy (MRS), and another subgroup (N = 305) IVGTTs. The clinical characteristics of the overall study group and the clamp, MRI/MRS, and IVGTT subgroups are presented in Table 1.

**Table 1:** Clinical characteristics of the study groups.

	Overall study group	Clampsubgroup	MRI/MRSsubgroup	IVGTTsubgroup	Myotubedonors TÜ	Myotubedonors ST
Sample size (N)	1,976	486	360	305	37	14
Women/men (%)	66.1/33.9	54.1/45.9	61.9/38.1	58.0/42.0	48.6/51.4	0/100
NGT/IFG/IGT/IFG&IGT/DIA (%)	70.4/11.3/9.8/8.5/0	75.7/7.4/10.1/6.8/0	63.1/12.2/13.6/11.1/0	65.9/10.5/14.1/9.5/0	91.9/0/5.4/0/2.7	57.1/0/0/0/42.9
Age (y)	40±13	40±12	45±12	45±11	28±7	62±4
BMI (kg/m^2^)	30.2±9.3	27.5±5.8	30.0±5.3	29.5±5.7	23.9±5.0	28.0±2.1
Body fat (%)	32.7±12.1	28.6±9.7	33.0±8.9	32.2±8.8	22.4±8.0	–
Waist circumference (cm)	96±19	93±15	97±14	97±15	82±11	–
Total adipose tissue (% BW)	–	–	30.5±9.1	–	–	–
Visceral adipose tissue (% BW)	–	–	3.33±1.74	–	–	–
Intrahepatic lipids (%)	–	–	5.88±6.43	–	–	–
Fasting glucose (mmol/L)	5.14±0.55	5.01±0.55	5.24±0.51	5.18±0.50	4.85±0.53	6.27±1.24
Glucose 120 min OGTT (mmol/L)	6.36±1.65	6.21±1.74	6.92±1.58	6.81±1.66	5.68±1.96	–
AUC_Ins 0–30_/AUC_Glc 0–30_OGTT (*10^–9^)	45.6±34.2	37.4±24.2	42.1±27.1	41.5±26.2	27.1±13.0	–
AUC_C-Pep 0–120_/AUC_Glc 0–120_OGTT (*10^–9^)	322±106	311±97	307±89	309±95	288±72	–
AIR IVGTT (pmol/L)	–	–	–	936±633	–	–
Fasting insulin (pmol/L)	71.3±61.0	53.7±39.1	63.8±42.4	61.6±42.2	45.2±26.1	67.8±37.1
HOMA-IR (*10^–6^ mol*U*L^–2^)	2.80±2.60	2.05±1.66	2.51±1.79	2.41±1.82	1.66±1.11	3.00±1.53
ISI OGTT (*10^15^ L^2^ *mol^–2^)	15.1±10.4	18.0±11.4	12.6±6.9	13.6±7.7	23.3±11.3	–
ISI Clamp (*10^6 ^L*kg^–1^ *min^–1^)	–	0.084±0.055	–	–	0.112±0.062*	–

Data are given as counts, percentages, or means ±SD. AIR – acute insulin response; AUC – area under the curve; BMI – body mass index; BW – body weight; C-Pep – C-peptide; DIA – diabetes; Glc – glucose; HOMA-IR – homeostasis model assessment of insulin resistance; IFG – impaired fasting glycaemia; IGT – impaired glucose tolerance; Ins – insulin; ISI – insulin sensitivity index; IVGTT – intravenous glucose tolerance test; MRI – magnetic resonance imaging; MRS – magnetic resonance spectroscopy; NGT – normal glucose tolerance; OGTT – oral glucose tolerance test; ST – Stockholm; TÜ – Tübingen;

* data available from 27 subjects.

#### OGTT

After a 10-h overnight fast, a standard 75-g OGTT was performed, and venous blood samples were drawn at time-points 0, 30, 60, 90, and 120 min for the determination of plasma glucose, insulin, and C-peptide concentrations [12].

#### IVGTT and hyperinsulinaemic-euglycaemic clamp

In those individuals who agreed to undergo both the IVGTT and the hyperinsulinaemic-euglycaemic clamp, the IVGTT was performed prior to the clamp after a 10-h overnight fast, as described by the Botnia protocol [13]. For the IVGTT, glucose (0.3 g/kg body weight) was given, and blood samples for the measurement of plasma glucose and insulin were obtained at time-points 0, 2, 4, 6, 8, 10, 20, 30, 40, 50, and 60 min [12]. For the hyperinsulinaemic-euglycaemic clamp, subjects received a primed infusion of insulin (40 mU*m^–2^*min^–1^) for 120 min, and glucose infusion was started to clamp the plasma glucose concentration at 5.5 mmol/L. Blood samples for the measurement of plasma glucose were obtained at 5-min intervals, plasma insulin levels were measured at baseline and in the steady state of the clamp [12]. In subjects who agreed to undergo the hyperinsulinaemic-euglycaemic clamp only, the clamp procedure was started after the 10-h overnight fast.

#### Measurements of body fat content and body fat distribution

Waist circumference (in cm) was measured in the upright position at the midpoint between the lateral iliac crest and the lowest rib. BMI was calculated as weight divided by height squared (kg/m^2^). The percentage of body fat was measured by bioelectrical impedance (BIA-101, RJL systems, Detroit, MI, USA). In addition, total and visceral fat contents (in % of body weight) were determined by whole-body MRI, as described earlier [14]. The intrahepatic lipid content (in % of signal) was determined by localized STEAM ^1^H-MRS, as formerly reported in detail [15].

#### Laboratory measurements

Plasma glucose (in mmol/L) was determined using a bedside glucose analyzer (glucose oxidase method, Yellow Springs Instruments, Yellow Springs, OH, USA). Plasma insulin and C-peptide concentrations (in pmol/L both) were measured by commercial chemiluminescence assays for ADVIA Centaur (Siemens Medical Solutions, Fernwald, Germany) according to the manufacturer’s instructions.

#### Calculations

HOMA-IR was calculated as {c(glucose[mmol/L])_0_*c(insulin[mU/L])_0_}/22.5 with c = concentration [16]. Therefore, HOMA-IR and fasting insulin concentrations are closely correlated (p<0.0001). The insulin sensitivity index derived from the OGTT (ISI OGTT) was estimated as proposed earlier [17]: 10,000/{c(glucose[mmol/L])_0_*c(insulin[pmol/L])_0_*c(glucose[mmol/L])_mean_*c(insulin[pmol/L])_mean_}^½^. The insulin sensitivity index derived from the hyperinsulinaemic-euglycaemic clamp (ISI clamp) was calculated as glucose infusion rate necessary to maintain euglycaemia during the last 20 min (steady state) of the clamp (in µmol*kg^–1^*min^–1^) divided by the steady-state insulin concentration (in pmol/L). OGTT-derived insulin release was estimated by AUC_Ins 0–30_/AUC_Glc 0–30_ and AUC_C-Pep 0–120_/AUC_Glc 0–120_ with Ins = insulin (in pmol/L), C-Pep = C-peptide (in pmol/L), and Glc = glucose (in mmol/L). AUC_Ins 0–30_/AUC_Glc 0–30_ was calculated as {c(insulin)_0_+c(insulin)_30_}/{c(glucose)_0_+c(glucose)_30_}. AUC_C-Pep 0–120_/AUC_Glc 0–120_ was calculated by the trapezoid method as ½{½c(C-peptide)_0_+c(C-peptide)_30_+c(C-peptide)_60_+c(C-peptide)_90_+½c(C-peptide)_120_}/½{½c(glucose)_0_+c(glucose)_30_+c(glucose)_60_+c(glucose)_90_+½c(glucose)_120_}. Both indices were recently shown to be superior to several fasting state−/OGTT-derived indices for the detection of genetically determined β-cell failure [18]. Acute insulin response (AIR) from the IVGTT was calculated according to the trapezoid method as ½{½c(insulin)_0_+c(insulin)_2_+c(insulin)_4_+c(insulin)_6_+c(insulin)_8_+½c(insulin)_10_}.

#### Selection of tagging SNPs

**Figure 1:**
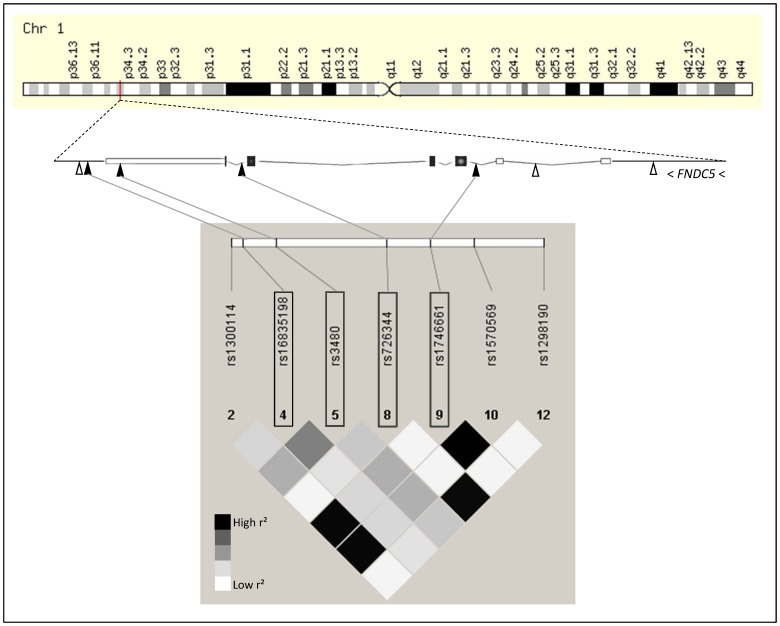
*FNDC5* gene locus on human chromosome 1p35.1 and tagging SNPs. The *FNDC5* gene consists of 6 exons and 5 introns and spans 8.47 kb from nucleotide position 33,100,464 to nucleotide position 33,108,934. The analyzed region additionally included 5 kb of the 5′-flanking region and 3 kb of the 3′-flanking region. This genomic region did not overlap with other known gene loci. The locations of the seven common (minor allele frequencies ≥0.05) SNPs in the region and the four tagging SNPs (highlighted by boxes) are indicated by white and black triangles, respectively. HapMap CEU-derived linkage disequilibrium data (r^2^-values) are presented as shaded diamonds (white – r^2^ = 0.0; black – r^2^ = 1.0; grey – in between). CEU – Central Europeans; SNP – single nucleotide polymorphism.

Based on publicly available phase III data of the International HapMap Project derived from the Central European (CEU) population (release #28 August 2010, http://hapmap.ncbi.nlm.nih.gov/index.html.en), we screened *in silico* a genomic area on human chromosome 1p35.1 encompassing the complete *FNDC5* gene (8.47 kb, 6 exons, 5 introns) as well as 5 and 3 kb of its 5′- and 3′-flanking regions, respectively (Figure 1). The *FNDC5* locus is flanked ∼16 kb upstream by the *HPCA* gene and ∼3.5 kb downstream by the *S100PBP* gene, but no high-linkage-disequilibrium blocks within the screened *FNDC5* locus region were found to overlap with these neighbouring genes, based on HapMap r^2^-data (Figure S1). Within the *FNDC5* locus, twelve HapMap SNPs were present and in Hardy-Weinberg equilibrium (HapMap data). Among these, eleven SNPs showed MAFs ≥0.05 (HapMap data), and one SNP, i.e., rs1284368, was rare (MAF = 0.004). As our study population is too small to assess rare variants with sufficient statistical power, we focussed on the common SNPs. Among the eleven common SNPs, only seven were genotyped in ≥50% of the HapMap individuals (HapMap CEU population: 46 family trios) and, thus, provide reliable data. All of these seven SNPs are located in non-coding regions of the locus. Their HapMap linkage disequilibrium (r^2^) data are schematically presented in Figure 1. Among these SNPs, four SNPs were selected as tagging SNPs covering all the other common SNPs within the locus with an r^2^ >0.8 (100% coverage) based on Tagger analysis using Haploview software (http://www.broadinstitute.org/scientific-community/science/programs/medical-and-population-genetics/haploview/haploview). As highlighted in Figure 1, the four tagging SNPs were rs16835198 (G/T) in the 3′-flanking region, rs3480 (A/G) in exon 6 (3′-untranslated region), rs726344 (G/A) in intron 5, and rs1746661 (G/T) in intron 2.

#### Genotyping

DNA was isolated from whole blood using a commercial DNA isolation kit (NucleoSpin, Macherey & Nagel, Düren, Germany). The four *FNDC5* tagging SNPs were genotyped using the Sequenom massARRAY system with iPLEX software (Sequenom, Hamburg, Germany). The genotyping success rates were ≥99.7%. The Sequenom results were validated by bidirectional sequencing in 50 randomly selected subjects, and both methods gave 100% identical results (r = 1.00).

#### Human myotube culture

Thirty-seven mostly young study participants (including two subjects with impaired glucose tolerance and one newly diagnosed treatment-naive diabetic patient) recruited in Tübingen and 14 elderly men (including 6 diabetic patients not under insulin treatment) recruited in Stockholm voluntarily agreed to undergo percutaneous needle biopsy of the vastus lateralis muscle (clinical characteristics of the donors presented in Table 1). From satellite cells that were obtained from the biopsies via collagenase digestion, primary human skeletal muscle cells were grown as formerly described in detail [19]. Basal gene expression was assessed in first-pass cells after growth to 80–90% confluence and five days of differentiation to myotubes [19]. The medium in which the myotubes were kept until cell lysis contained 2% fetal calf serum and 1 mg/L glucose.

#### Quantitative PCR (qPCR)

Myotubes were washed and harvested by trypsinization. RNA was isolated with RNeasy columns (Qiagen, Hilden, Germany). Total RNA treated with RNase-free DNase I was transcribed into cDNA using AMV reverse transcriptase and the First Strand cDNA kit from Roche Diagnostics (Mannheim, Germany). QPCR was performed in duplicates with fluorescence-labelled probes from Roche Universal ProbeLibrary on a LightCycler™ (Roche Diagnostics, Mannheim, Germany). Primers were purchased from TIB MOLBIOL (Berlin, Germany). Primer sequences and PCR conditions are available upon request. All quantitative mRNA data were normalized to the housekeeping gene *RPS13* using the ΔC_t_ method.

#### Statistical analyses

Hardy-Weinberg equilibrium was tested using χ^2^ test (one degree of freedom). Linkage disequilibrium (D’, r^2^) between the tagging SNPs was analysed using MIDAS 1.0 freeware (http://www.genes.org.uk/software/midas, [20]). Continuous variables with non-normal distribution were log*_e_*-transformed prior to linear regression analysis. Multiple linear regression analysis was performed using the least-squares method. In the regression models, the trait of interest (measure of body fat content/distribution, glycaemia, insulin sensitivity, or insulin release) was chosen as outcome variable, the SNP genotype (in the additive inheritance model) as independent variable, and gender, age, body fat content/BMI as possible confounding variables. Based on screening four non-linked tagging SNPs in parallel, a p-value <0.0127 was considered statistically significant according to Bonferroni correction for multiple comparisons. We did not correct for the tested metabolic traits since these were far from being independent. In all subsequent analyses addressing exclusively the effects of SNPs rs16835198 and rs726344 on insulin sensitivity in more detail, a p-value <0.0253 was considered statistically significant. We did this because we assumed that the chance to get a statistical chance finding in a hypothesis-driven replication effort in the absence of multiple testing is extremely low. For all these analyses, the statistical software package JMP 10.0 (SAS Institute, Cary, NC, USA) was used. The effects of SNPs rs726344 and rs16835198 on insulin sensitivity in our TÜF-derived overall study group and in the Meta-Analyses of Glucose and Insulin-related traits Consortium (MAGIC) was studied by inverse variance weighted meta-analysis using MetaXL freeware (http://www.epigear.com/index_files/metaxl.html). Our study was sufficiently powered (1-β≥0.8) to detect effect sizes between 6.2% (rs3480) and 10% (rs726344) on ISI OGTT (two-sided type 1 error rate <0.05). Power calculations were performed using Quanto 1.2.4 freeware (http://hydra.usc.edu/gxe). For gene expression studies, t-tests, simple and multiple linear regression analyses were applied wherever appropriate, and the significance threshold was set to p≤0.05.

## Results

### 

#### Clinical characteristics of the study groups

The overall study group (N = 1,976) consisted of relatively young (median age –39 y) and moderately overweight (median BMI –27.6 kg/m^2^) non-diabetic individuals with a proportion of 66% being female and a proportion of 34% being male. The majority (∼70%) of the subjects were normal glucose tolerant (NGT), ∼30% were prediabetic: 11.3% had isolated impaired fasting glycaemia (IFG), 9.8% isolated impaired glucose tolerance (IGT), and 8.5% both IFG and IGT. The clinical characteristics of the study participants are presented in Table 1. The clinical characteristics of the clamp, MRI/MRS, and IVGTT subgroups were largely comparable (Table 1).

#### Genotyping of *FNDC5* tagging SNPs

The 1,976 study participants were genotyped for the four tagging SNPs rs16835198, rs3480, rs726344, and rs1746661 covering all other common variants in the *FNDC5* gene locus with MAFs ≥0.05 (Figure 1). The genotyping success rates were ≥99.7%, and three tagging SNPs obeyed the Hardy-Weinberg equilibrium (p≥0.2, Table 2). SNP rs1746661 significantly deviated from Hardy-Weinberg equilibrium (p = 0.0292, Table 2). Since no genotyping errors could be detected, we included this SNP in our analyses. The MAFs observed in our overall study group ranged from 0.10 to 0.42 and were close to those reported for the HapMap CEU population (Table S1). Based on r^2^ data, the observed genetic linkage between the tagging SNPs was low or moderate (r^2^ range –0.03–0.50, Table S2).

**Table 2:** Association of *FNDC5* SNPs rs16835198, rs3480, rs726344, and rs1746661 with glycaemia and insulin sensitivity (statistics).

SNP	Genotype	N Overallstudy group	HWE	Fasting glucose(mmol/L)	Glucose 120 minOGTT (mmol/L)	Fasting insulin(pmol/L)	HOMA-IR(*10^–6^ mol*U*L^–2^)	ISI OGTT(*10^15^ L^2^*mol^–2^)	N Clampsubgroup	ISI Clamp(*10^6^ L*kg^–1^*min^–1^)
rs16835198	GG/GT/TT	844/892/238	p = 0.9	β = –0.0003 p = 0.9	β = 0.0005 p = 1.0	**β = –0.0457 p = 0.0118** #	**β = –0.0459 p = 0.0179**	**β = 0.0480 p = 0.0126** #	209/221/55	β = 0.0081 p = 0.8
rs3480	AA/AG/GG	689/928/355	p = 0.2	β = 0.0012 p = 0.7	β = 0.0007 p = 0.9	β = 0.0281 p = 0.1	β = 0.0294 p = 0.1	β = –0.0286 p = 0.1	159/240/86	β = 0.0022 p = 0.9
rs726344	GG/GA/AA	1,590/359/22	p = 0.7	**β = 0.0111 p = 0.0281**	β = 0.0071 p = 0.6	**β = 0.0708 p = 0.0131**	**β = 0.0817 p = 0.0073** #	**β = –0.0809 p = 0.0074** #	381/94/9	β = 0.0778 p = 0.1
rs1746661	GG/GT/TT	1,240/627/105	p = 0.0292	β = –0.0010 p = 0.8	β = 0.0050 p = 0.6	β = 0.0243 p = 0.2	β = 0.0237 p = 0.3	β = –0.0094 p = 0.7	304/151/30	β = –0.0361 p = 0.3

Prior to statistical analysis, all parameters were adjusted for gender, age, and bioelectrical impedance-derived percentage of body fat. Nominal associations are marked by bold fonts;

# significant after Bonferroni correction (p<0.0127). HOMA-IR – homeostasis model assessment of insulin resistance; HWE – Hardy-Weinberg equilibrium; ISI – insulin sensitivity index; OGTT – oral glucose tolerance test; SNP – single nucleotide polymorphism.

#### Genetic associations of *FNDC5* with body fat content and body fat distribution

After adjustment for gender and age, none of the four tagging SNPs showed significant or nominal association (p≥0.1, Table S3) with parameters of body fat content (BMI, bioelectrical impedance-derived percentage of body fat, MRI-derived total adipose tissue mass) or body fat distribution (waist circumference, MRI-derived visceral adipose tissue mass, MRS-derived intrahepatic lipids).

#### Genetic associations of *FNDC5* with insulin release

After adjustment for gender, age, bioelectrical impedance-derived percentage of body fat, and ISI OGTT, none of the tagging SNPs was significantly or nominally associated with OGTT-derived parameters of insulin release (p≥0.6) or with IVGTT-derived AIR (p≥0.5) as given in Table S4.

#### Genetic associations of *FNDC5* with insulin sensitivity and glycaemia

**Figure 2:**
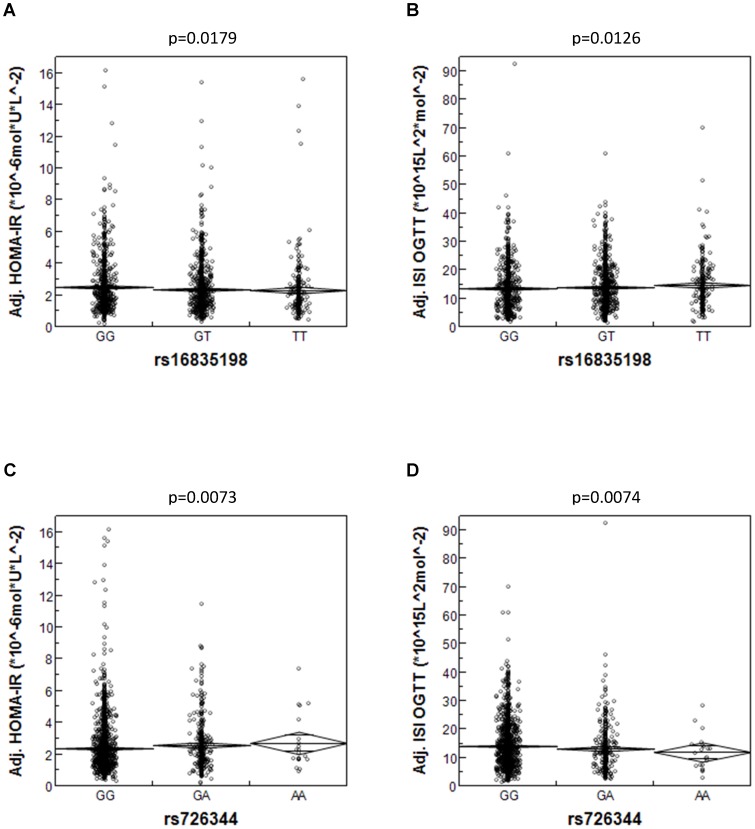
Association of *FNDC5* SNPs rs16835198 and rs726344 with insulin sensitivity. HOMA-IR (A and C) and ISI OGTT (B and D) data were adjusted for gender, age, and bioelectrical impedance-derived percentage of body fat. Diamonds represent means ±SE. HOMA-IR – homeostasis model assessment of insulin resistance; ISI OGTT – oral glucose tolerance test-derived insulin sensitivity index; SNP – single nucleotide polymorphism.

After adjustment for gender, age, and percentage of body fat, the major G-allele of SNP rs16835198 was significantly associated with elevated fasting insulin concentrations (p = 0.0118) and reduced ISI OGTT (p = 0.0126) and nominally associated with increased HOMA-IR (p = 0.0179) revealing an additive insulin-desensitizing effect of this allele (raw data shown in Table S5, adjusted data shown in Figure 2A and B, statistics shown in Table 2). After identical adjustment, the minor A-allele of SNP rs726344 was significantly associated with increased HOMA-IR (p = 0.0073) and reduced ISI OGTT (p = 0.0074) and nominally associated with increased fasting insulin concentrations (p = 0.0131) demonstrating an additive insulin-desensitizing effect of this allele (raw data shown in Table S5, adjusted data shown in Figure 2C and D, statistics shown in Table 2). Furthermore, the insulin-desensitizing allele of rs726344 was nominally associated with increased fasting glucose concentrations (p = 0.0281, Table 2, raw data shown in Table S5). None of the other tested SNPs showed associations with insulin sensitivity and/or glycaemia. To test whether the effects of SNP rs16835198 were mediated by the weakly linked SNP rs726344 (r^2^ = 0.061, Table 2) and vice versa, we performed conditional analyses. After adjustment of SNP rs16835198 for gender, age, percentage of body fat and SNP rs726344, the associations of SNP rs16835198 with fasting insulin concentrations and ISI OGTT were still nominal (p = 0.0424 and p = 0.0496, respectively), whereas its association with HOMA-IR was no longer nominal (p = 0.07). The associations of SNP rs726344 with fasting glucose concentrations, fasting insulin concentrations, HOMA-IR, and ISI OGTT were still nominal after additional adjustment for SNP rs16835198 (p = 0.0252, p = 0.0497, p = 0.0275, and p = 0.0298, respectively) pointing to independent effects of both SNPs and weaker effects of SNP rs16835198. Furthermore, both SNPs provided divergent results in NGT vs. prediabetic (sum of IFG, IGT, and IFG+IGT) subjects: the effect of SNP rs16835198 on insulin sensitivity (as assessed by fasting insulin concentrations, HOMA-IR, and ISI OGTT) was present in NGT (β≥0.0492, p≤0.0216), but not in prediabetic (β≤0.0216, p≥0.5), subjects, whereas the effect of SNP rs726344 emerges in prediabetic (β≥0.0875, p≤0.09), but not in NGT (β≤0.0490, p≥0.1), subjects. The effect of SNP rs726344 on fasting glucose concentrations was detectable in prediabetic subjects only (β = 0.0210, p = 0.0131; NGT subjects: β = 0.0029, p = 0.6).

#### Interrogation of MAGIC data for replication

**Figure 3:**
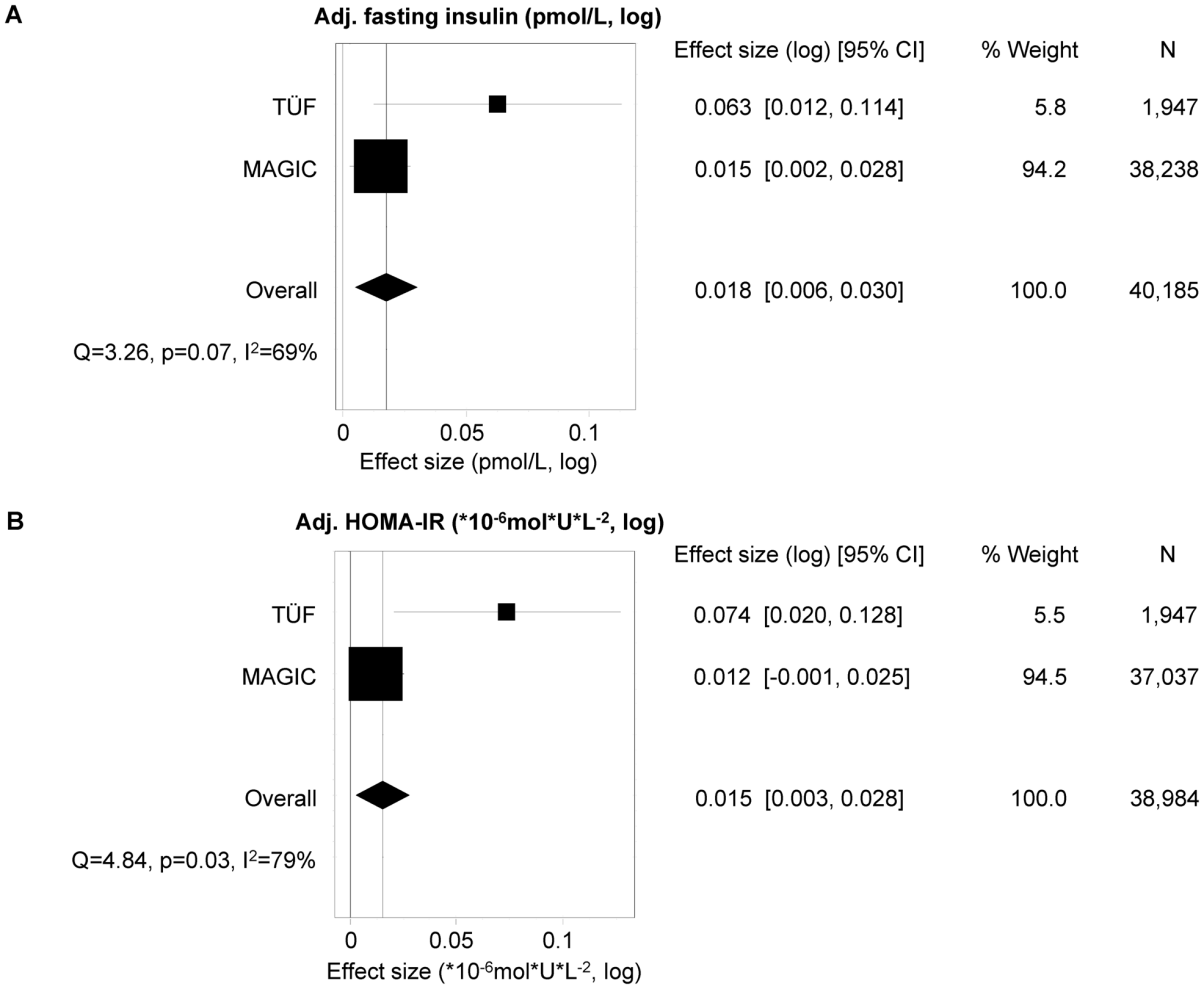
Meta-analysis of the effect of *FNDC5* SNP rs726344 on insulin sensitivity in TÜF and MAGIC. The effects of the minor A-allele of SNP rs726344 on fasting insulin (A) and HOMA-IR (B), as derived from multiple linear regression analysis with gender, age, and BMI as confounding variables, were subjected to inverse variance weighted meta-analysis. Effect sizes, 95% confidence intervals, weights, sample sizes, and heterogeneity data are given. HOMA-IR – homeostasis model assessment of insulin resistance; MAGIC – Meta-Analyses of Glucose and Insulin-related traits Consortium; SNP – single nucleotide polymorphism; TÜF – overall study group derived from the Tübingen Family study for type 2 diabetes.

To replicate the effects of SNPs rs16835198 and rs726344 on fasting insulin concentrations and HOMA-IR, we screened the publicly available MAGIC data from 38,238 (fasting insulin dataset) and 37,037 (HOMA-IR dataset) subjects (http://www.magicinvestigators.org/downloads, [21]) and found a concordant and significant association of the A-allele of SNP rs726344 with elevated fasting insulin concentrations (p = 0.01669) and a non-significant trend for association with increased HOMA-IR (p = 0.08). SNP rs16835198 was not associated with either parameter in MAGIC (p≥0.7). To further corroborate the effect of SNP rs726344 on insulin sensitivity, we meta-analysed the effects on fasting insulin and HOMA-IR reported for this SNP’s A-allele in MAGIC and the effects of the A-allele derived from comparably performed multiple linear regression models in our overall study group. In the meta-analysis, the effect sizes of the A-allele were shifted to higher (and more significant) values compared to those reported by MAGIC (fasting insulin –0.018 vs. 0.015, p = 0.0002; HOMA-IR –0.015 vs. 0.012, p = 0.0015), as depicted in Figure 3. As expected from the MAGIC data alone, meta-analysis did not reveal significant effects of SNP rs16835198 on insulin sensitivity (Figure S2).

#### Association of human myotube *FNDC5* expression with insulin sensitivity

**Figure 4:**
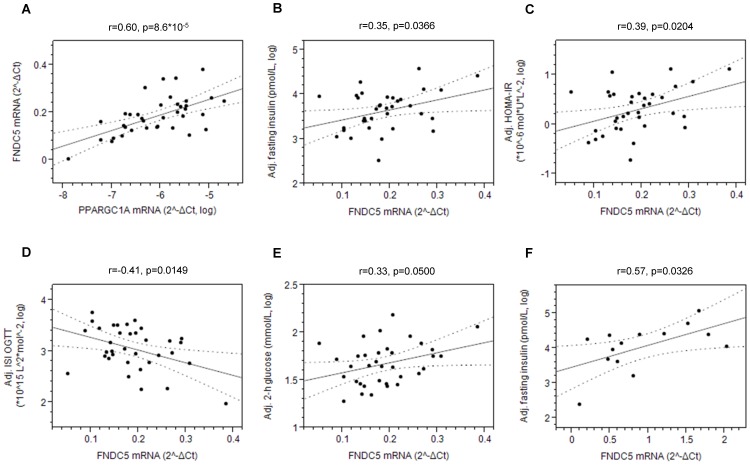
Association of human myotube *FNDC5* mRNA expression with *PPARGC1A* mRNA expression *in vitro* and donors’ insulin sensitivity *in vivo*. The association between human myotube *FNDC5* and *PPARGC1A* mRNA contents (A) was assessed using simple linear regression analysis. The association between human myotube *FNDC5* mRNA expression and fasting insulin levels (B), HOMA-IR (C), ISI OGTT (D), and 2-h plasma glucose levels (E) of 37 young healthy donors recruited in Tübingen and with fasting insulin levels (F) of 14 elderly men recruited in Stockholm was tested by multiple linear regression analysis with gender, age, and bioelectrical impedance-derived percentage of body fat (Tübingen volunteers) or with BMI (Stockholm volunteers) as confounding variables (leverage plots shown). Dotted lines indicate the 95% confidence interval of the regression. HOMA-IR – homeostasis model assessment of insulin resistance.

To further address the role of FNDC5/irisin in humans, we quantified *FNDC5* mRNA expression in myotubes derived from 37 mostly young participants (Table 1) of the overall study group. This gene’s basal expression levels were not influenced by donors’ gender, age, or percentage of body fat (p≥0.3, Figure S3). Then, we addressed whether we can replicate, in human myotubes, the close association between PGC-1α and FNDC5 that was observed in mice upon muscle-specific transgenic PGC-1α overexpression [10]. As depicted in Figure 4A, the basal *PPARGC1A* (encoding PGC-1α) and *FNDC5* mRNA contents of human myotubes were closely associated (r = 0.60, p = 8.6*10^–5^). Based on our SNP data, we finally asked whether myotube *FNDC5* expression is associated with *in vivo* insulin sensitivity of the donors. In contrast to the findings in mice, i.e., insulin sensitization of high fat-fed mice upon adenoviral *Fndc5* overexpression [10], basal *FNDC5* expression in human myotubes was positively associated with fasting insulin concentrations (p = 0.0366, Figure 4B) and HOMA-IR (p = 0.0204, Figure 4C), negatively associated with ISI OGTT (p = 0.0149, Figure 4D), and positively associated with 2-h glucose concentrations (p = 0.0500, Figure 4E) after adjustment of the metabolic trait for gender, age, and percentage of body fat. Even though there was a weak trend for association of the insulin-desensitizing minor A-allele of *FNDC5* SNP rs726344 with higher *FNDC5* mRNA contents (p = 0.19), this SNP’s MAF was too low to allow a reliable evaluation (only four heterozygous and no homozygous carriers of the minor allele were present among the myotube donors).

#### Replication of the *in vitro* results

To this end, we determined *FNDC5* expression in human myotubes from 14 elderly men (8 normal glucose tolerant subjects, 6 diabetic patients; Table 1) recruited at the Karolinska Institute in Stockholm. Importantly, none of the diabetic patients was under insulin treatment. From these donors, only age, BMI, and fasting glucose and insulin concentrations were available. Again, the basal *FNDC5* expression levels were not influenced by donors’ age or BMI (p≥0.5). After adjustment for BMI, basal *FNDC5* expression was positively associated with fasting insulin levels (p = 0.0326, Figure 4F) and showed a trend towards positive association with HOMA-IR (p = 0.06). In further support of our data, a recent report by Timmons et al. also provided a trend for positive association between *FNDC5* expression in freshly isolated skeletal muscle biopsies (without isolation and culture of myocytes) from 118 diabetes medication-free subjects and donors’ fasting insulin levels (r = 0.2, not significant [22]).

## Discussion

In this study, we report a significant and replicated insulin-desensitizing effect of the minor A-allele of *FNDC5* SNP rs726344 (adjusted effect size on HOMA-IR in our study population – +9.5% per A-allele). Since this SNP and the only HapMap SNP reported to be in high linkage with it, i.e., rs1298190 (r^2^ = 0.96, Figure 1), are both intronic, the molecular mechanisms how these SNPs affect insulin sensitivity remain obscure. Unfortunately, we were not able to reliably study this SNP’s impact on *FNDC5* expression in our myotube donors due to the SNP’s low MAF ( = 0.10).

The replicated finding that *FNDC5* expression is inversely associated with the donors’ insulin sensitivity appears conflicting with the mouse data from Boström et al. who reported reduced insulin resistance in high fat-fed mice upon adenoviral *Fndc5* overexpression via (‘browning’-mediated) elevated energy expenditure and attenuated weight gain [10]. The reasons for this discrepancy may be diverse. Even though it was recently convincingly demonstrated that functional brown adipose tissue exists in adult humans [23]–[26], it is currently unclear whether ‘browning’, i.e., trans-determination and/or trans-differentiation of human white adipose precursor cells into brown adipocytes occurs in humans *in vivo*, as was shown in mice [10], [27]–[29]. Moreover, it is completely unknown whether the FNDC5-derived myokine irisin exerts similar biological functions in mice and humans, and mice and humans may differ, e.g., in the regulation of FNDC5′s post-translational processing (glycosylation, protease-stimulated cleavage) and/or in the regulation of cellular irisin release. Interestingly and in very good agreement with our results, a positive association between irisin plasma levels and fasting insulin levels, as a rough estimate of insulin resistance, was very recently demonstrated by Stengel et al. [30]. Moreover, Timmons et al. [22] could not establish irisin as an exercise factor in humans, but this was shown by Boström et al. in mice. Thus, irisin’s role in humans is far from being understood, and there are several lines of evidence for species-specific differences between mice and humans.

Our translational data showing an association between myotube *FNDC5* expression and insulin sensitivity of the donors imply that *FNDC5* expression in vivo is maintained during muscle biopsy, isolation of stellate cells, and in vitro differentiation to myotubes. Since we observed similar associations between *ANGPTL4*, *PDK4*, *SCD*, and *ADIPOR1* expression in human myotubes and in vivo traits of the donors earlier [3], [31]–[33], we suggest that the expression of a series of genes is indeed stable, and this may have genetic and/or epigenetic reasons.

Notably, we identified a second *FNDC5* SNP, i.e., rs16835198, with markedly weaker, but significant, effects on parameters of insulin sensitivity (adjusted effect size on HOMA-IR in our study population –4.7% per major G-allele). This SNP is located in the 3′-flanking region of the gene and was in rather low linkage with SNP rs726344 (r^2^ = 0.061). Furthermore, both SNPs exerted independent effects on insulin sensitivity and revealed divergent effects on insulin sensitivity in NGT vs. prediabetic subjects. The latter finding, however, has to be interpreted with caution due to the limited sample sizes of the subgroups (NGT subjects: N = 1,392; prediabetic subjects: N = 584), but could point to SNP-specific genotype-glycaemia interactions. This clearly needs deeper examination in larger study populations. In contrast to rs726344, this SNP’s major allele revealed an insulin-desensitizing effect. This difference could be, for instance, explained by transcription rate-attenuating versus -enhancing effects of these two rather independent nucleotide exchanges. To assess whether the SNPs indeed affect the transcription rate and transcription factor binding sites in enhancer/silencer elements, further functional studies are needed.

Notably, both SNPs rs726344 and rs16835198 revealed smaller effect sizes on fasting insulin and HOMA-IR in MAGIC as compared to TÜF and the effect of SNP rs16835198 was no longer significant in MAGIC. One explanation for this observation may be the greater heterogeneity of MAGIC genome-wide association studies, e.g., in measured insulin values. In our experience, the method of insulin measurement is one of the most critical points whenever insulin data have to be compared between different studies.

An intriguing finding of our study is the lack of association of *FNDC5* SNPs rs726344 and rs16835198 with hyperinsulinaemic-euglycaemic clamp-derived insulin sensitivity. This may reflect the limited statistical power of the substantially smaller clamp subgroup. On the other hand, this could also be due to organ-specific insulin-desensitizing effects of irisin that are better detected by fasting- and OGTT-derived measures of insulin sensitivity. In this regard, it has been suggested that HOMA-IR and the OGTT-derived insulin sensitivity index used in this study are proxies reflecting, to a large part, hepatic insulin sensitivity, whereas hyperinsulinaemic-euglycaemic clamp-derived insulin sensitivity indices measure whole-body insulin sensitivity [34], [35]. Clearly, this issue needs further investigation, e.g., by measurement of organ-specific insulin sensitivity via tracer methods [36].

A limitation of the study could be that we applied Bonferroni correction of the significance threshold for the four non-linked tagging SNPs only. We did not perform additional correction for the four prediabetic phenotypes tested, i.e., overweight, glucose intolerance, insulin resistance, and impaired insulin release, since these traits are far from being independent, and testing highly dependent traits is well known to result in actual error rates far below the adjusted error rates. A more rigorous correction, at the costs of an increasing number of statistical type II errors, would have rendered most of our significant results nominal. The fact that we identified two non-linked SNPs within the same locus – and not just a single one – both with effects on insulin sensitivity, but not on body adiposity or insulin secretion, further argues against mere chance findings.

In conclusion, this study provides evidence that the *FNDC5* gene, encoding the novel myokine irisin, influences insulin sensitivity in humans. Our gene expression data revealed an unexpected and currently inexplicable insulin-desensitizing effect of irisin. Based on this finding, it would now be interesting to study this gene’s impact on type 2 diabetes risk.

## Supporting Information

Figure S1 **Linkage disequilibrium structure of the 200-kb genomic region surrounding the** ***FNDC5*** **gene.** Genes (with exon-intron structure) are written in red colour. *FNDC5* is marked by yellow shading. HapMap CEU-derived linkage disequilibrium data (r^2^-values) are presented as shaded diamonds (white – r^2^ = 0.0; black – r^2^ = 1.0; grey – in between). CEU – Central Europeans.
(TIFF)

Figure S2 **Meta-analysis of the effect of** ***FNDC5*** **SNP rs16835198 on insulin sensitivity in TÜF and MAGIC.** The effects of the major G-allele of SNP rs16835198 on fasting insulin (A) and HOMA-IR (B), as derived from multiple linear regression analysis with gender, age, and BMI as confounding variables, were subjected to inverse variance weighted meta-analysis. Effect sizes, 95% confidence intervals, weights, sample sizes, and heterogeneity data are given. HOMA-IR – homeostasis model assessment of insulin resistance; MAGIC – Meta-Analyses of Glucose and Insulin-related traits Consortium; SNP – single nucleotide polymorphism; TÜF – overall study group derived from the Tübingen Family study for type 2 diabetes.
(TIFF)

Figure S3 **Association of human myotube** ***FNDC5*** **mRNA expression with donors’ gender, age, and body fat content.** The association between human myotube *FNDC5* mRNA contents and donors’ gender (A) was assessed by Student’s t-test. The association between human myotube *FNDC5* mRNA expression and donors’ age (B) and body fat content (C) was tested by multiple linear regression analysis. Dotted lines indicate the 95% confidence interval of the regression.
(TIFF)

Table S1 **Minor allele frequencies of** ***FNDC5*** **tagging SNPs.** CEU – Central Eurpeans; SNP – single nucleotide polymorphism.
(DOCX)

Table S2 **Linkage disequilibrium between** ***FNDC5*** **tagging SNPs.** Data represent linkage disequilibrium data: D’ values are given below empty cell, r^2^ values above empty cells. CEU – Central Europeans; SNP – single nucleotide polymorphism.
(DOCX)

Table S3 **Association of** ***FNDC5*** **SNPs rs16835198, rs3480, rs726344, and rs1746661 with body fat content and body fat distribution.** Data are shown as unadjusted raw data (means ±SD). Prior to statistical analysis, all parameters were adjusted for gender and age. BMI – body mass index; BW – body weight; MRI – magnetic resonance imaging; MRS – magnetic resonance spectroscopy; SNP – single nucleotide polymorphism.
(DOCX)

Table S4 **Association of** ***FNDC5*** **SNPs rs16835198, rs3480, rs726344, and rs1746661 with insulin release.** Data are shown as unadjusted raw data (means ±SD). Prior to statistical analysis, all parameters were adjusted for gender, age, percentage of body fat, and OGTT-derived insulin sensitivity. AIR – acute insulin response; AUC – area under the curve; C-Pep – C-peptide; Glc – glucose; Ins – insulin; IVGTT – intravenous glucose tolerance test; OGTT – oral glucose tolerance test; SNP – single nucleotide polymorphism.
(DOCX)

Table S5 **Association of** ***FNDC5*** **SNPs rs16835198, rs3480, rs726344, and rs1746661 with glycaemia and insulin sensitivity (raw data).** Data are shown as unadjusted raw data (means ±SD). HOMA-IR – homeostasis model assessment of insulin resistance; ISI – insulin sensitivity index; OGTT – oral glucose tolerance test; SNP – single nucleotide polymorphism.
(DOCX)
