# Supplementary figures and images for: Common Genetic Variation in the Human FNDC5 Locus, Encoding the Novel Muscle-Derived ‘Browning’ Factor Irisin, Determines Insulin Sensitivity
Source: PLoS One. 2013 Apr 25;8(4):e61903. doi: 10.1371/journal.pone.0061903 (PMC3636229; doi:10.1371/journal.pone.0061903)

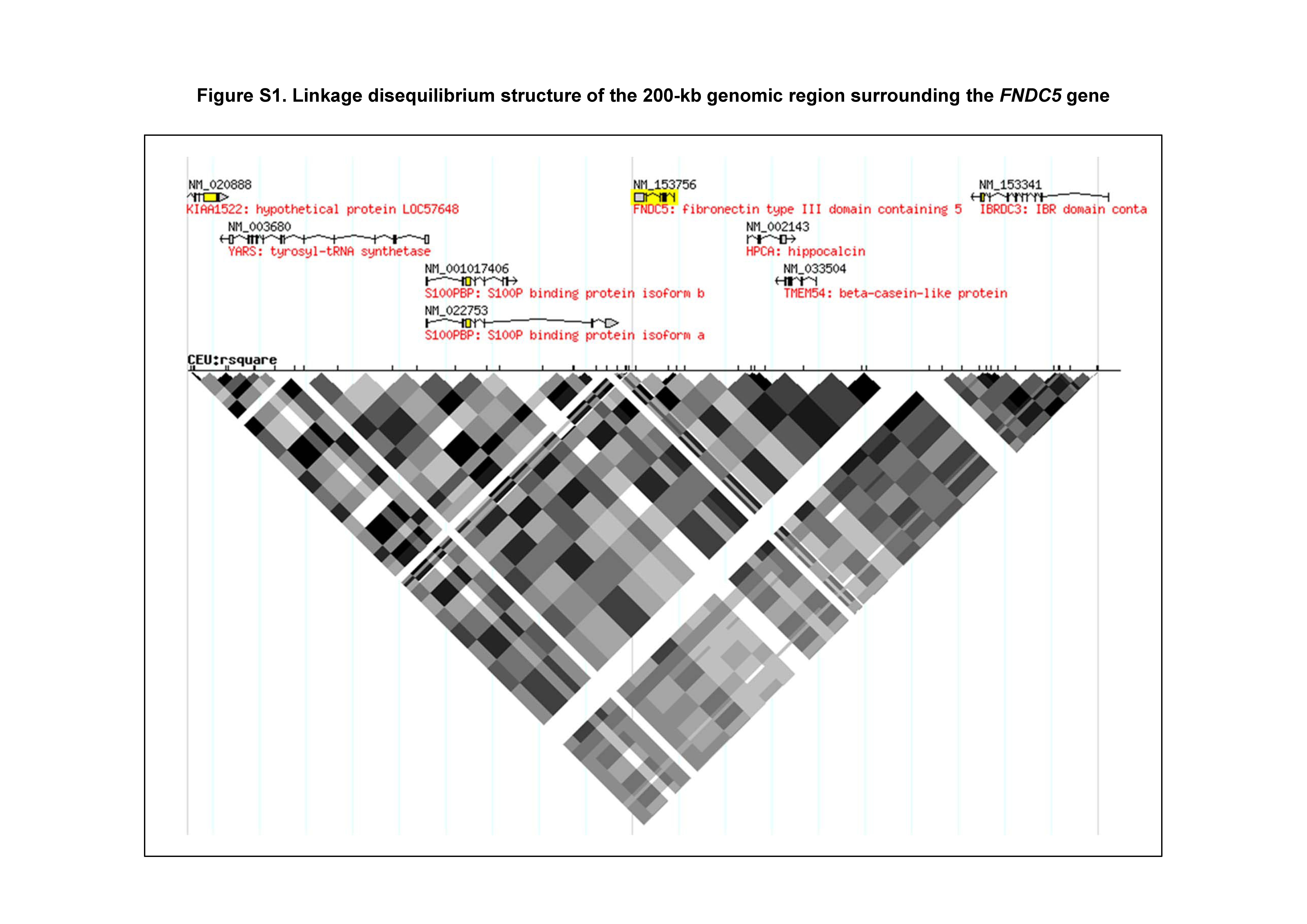

Supplement: Figure S1 — Linkage disequilibrium structure of the 200-kb genomic region surrounding the FNDC5 gene. Genes (with exon-intron structure) are written in red colour. FNDC5 is marked by yellow shading. HapMap CEU-derived linkage disequilibrium data (r2-values) are presented as shaded diamonds (white – r2 = 0.0; black – r2 = 1.0; grey – in between). CEU – Central Europeans. (TIFF) [file pone.0061903.s001.tiff]

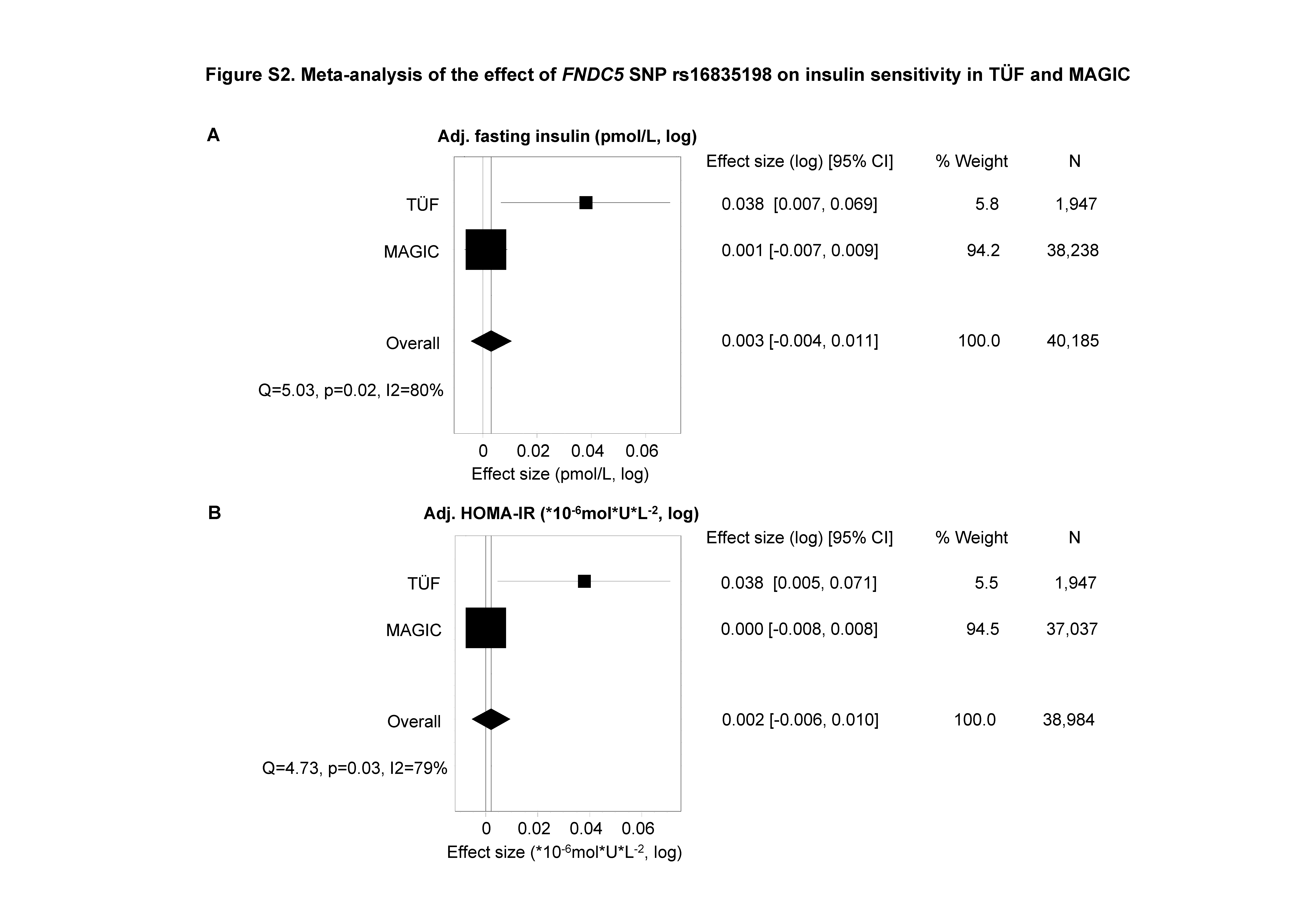

Supplement: Figure S2 — Meta-analysis of the effect of FNDC5 SNP rs16835198 on insulin sensitivity in TÜF and MAGIC. The effects of the major G-allele of SNP rs16835198 on fasting insulin (A) and HOMA-IR (B), as derived from multiple linear regression analysis with gender, age, and BMI as confounding variables, were subjected to inverse variance weighted meta-analysis. Effect sizes, 95% confidence intervals, weights, sample sizes, and heterogeneity data are given. HOMA-IR – homeostasis model assessment of insulin resistance; MAGIC – Meta-Analyses of Glucose and Insulin-related traits Consortium; SNP – single nucleotide polymorphism; TÜF – overall study group derived from the Tübingen Family study for type 2 diabetes. (TIFF) [file pone.0061903.s002.tiff]

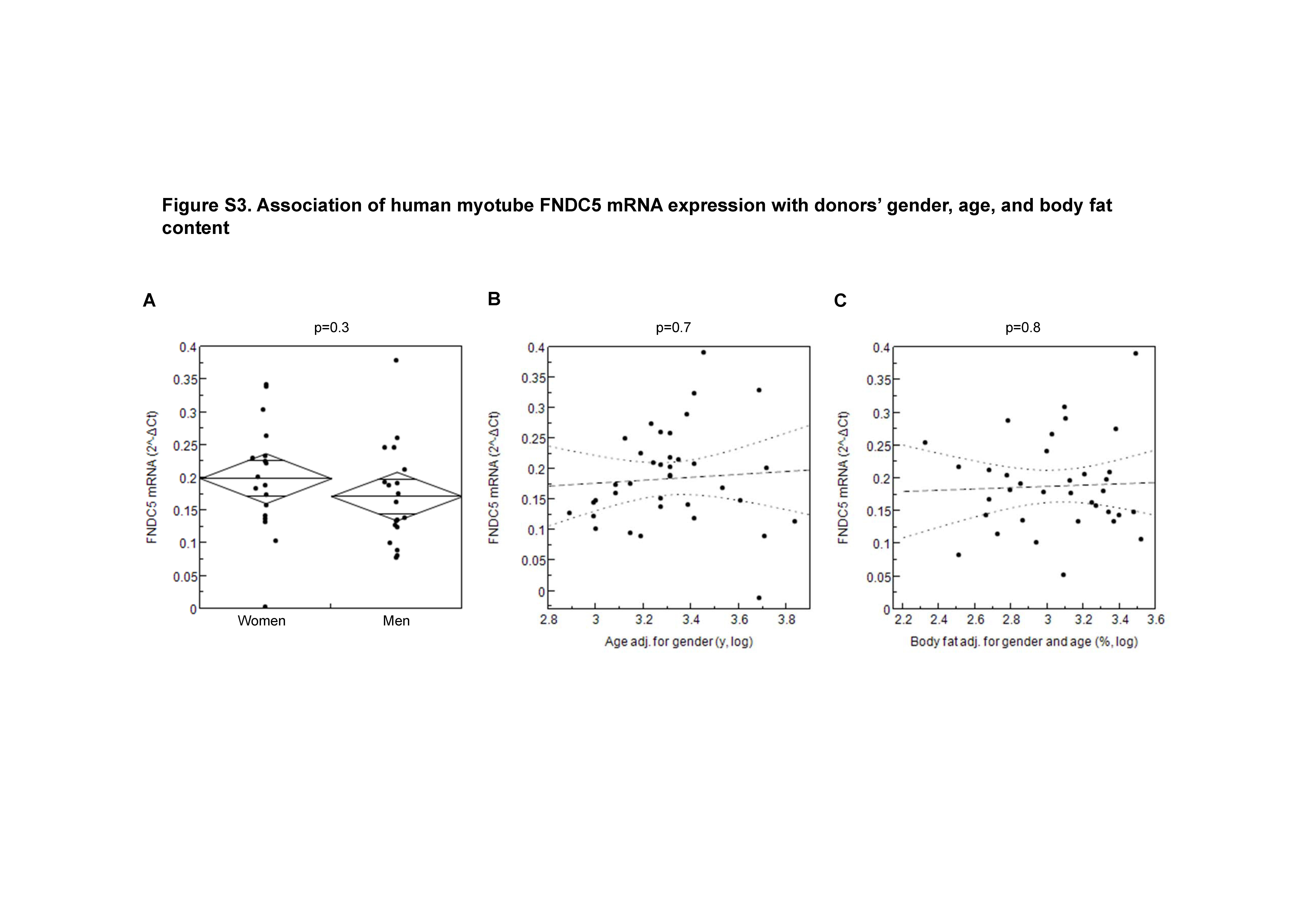

Supplement: Figure S3 — Association of human myotube FNDC5 mRNA expression with donors’ gender, age, and body fat content. The association between human myotube FNDC5 mRNA contents and donors’ gender (A) was assessed by Student’s t-test. The association between human myotube FNDC5 mRNA expression and donors’ age (B) and body fat content (C) was tested by multiple linear regression analysis. Dotted lines indicate the 95% confidence interval of the regression. (TIFF) [file pone.0061903.s003.tiff]
