# Supplementary material for: Common Genetic Variation in the Human FNDC5 Locus, Encoding the Novel Muscle-Derived ‘Browning’ Factor Irisin, Determines Insulin Sensitivity
Source: PLoS One. 2013 Apr 25;8(4):e61903. doi: 10.1371/journal.pone.0061903 (PMC3636229; doi:10.1371/journal.pone.0061903)
Supplement: Table S1 — Minor allele frequencies of FNDC5 tagging SNPs. CEU – Central Eurpeans; SNP – single nucleotide polymorphism. (DOCX) [file pone.0061903.s004.docx]

**Table S1. Minor allele frequencies of *FNDC5* tagging SNPs**

| SNP | HapMap CEU | Own overall study group |
| --- | --- | --- |
| rs16835198 | 0.407 | 0.347 |
| rs3480 | 0.416 | 0.415 |
| rs726344 | 0.137 | 0.102 |
| rs1746661 | 0.185 | 0.212 |

CEU – Central Eurpeans; SNP – single nucleotide polymorphism
