# Supplementary material for: Common Genetic Variation in the Human FNDC5 Locus, Encoding the Novel Muscle-Derived ‘Browning’ Factor Irisin, Determines Insulin Sensitivity
Source: PLoS One. 2013 Apr 25;8(4):e61903. doi: 10.1371/journal.pone.0061903 (PMC3636229; doi:10.1371/journal.pone.0061903)
Supplement: Table S2 — Linkage disequilibrium between FNDC5 tagging SNPs. Data represent linkage disequilibrium data: D’ values are given below empty cell, r2 values above empty cells. CEU – Central Europeans; SNP – single nucleotide polymorphism. (DOCX) [file pone.0061903.s005.docx]

**Table S2. Linkage disequilibrium between *FNDC5* tagging SNPs**

| HapMap CEU | | | | |  | Own overall study group | | | | |
| --- | --- | --- | --- | --- | --- | --- | --- | --- | --- | --- |
|  | rs16835198 | rs3480 | rs726344 | rs1746661 |  | rs16835198 | rs3480 | rs726344 | rs1746661 |  |
| rs16835198 | − | 0.498 | 0.107 | 0.155 |  | − | 0.374 | 0.061 | 0.140 | rs16835198 |
| rs3480 | 1.0 | − | 0.214 | 0.316 |  | 1.0 | − | 0.161 | 0.375 | rs3480 |
| rs726344 | 1.0 | 1.0 | − | 0.041 |  | 1.0 | 1.0 | − | 0.031 | rs726344 |
| rs1746661 | 1.0 | 1.0 | 1.0 | − |  | 1.0 | 1.0 | 1.0 | − | rs1746661 |

Data represent linkage disequilibrium data: D’ values are given below empty cell, r² values above empty cells. CEU – Central Europeans; SNP – single nucleotide polymorphism
