# Supplementary material for: Common Genetic Variation in the Human FNDC5 Locus, Encoding the Novel Muscle-Derived ‘Browning’ Factor Irisin, Determines Insulin Sensitivity
Source: PLoS One. 2013 Apr 25;8(4):e61903. doi: 10.1371/journal.pone.0061903 (PMC3636229; doi:10.1371/journal.pone.0061903)
Supplement: Table S3 — Association of FNDC5 SNPs rs16835198, rs3480, rs726344, and rs1746661 with body fat content and body fat distribution. Data are shown as unadjusted raw data (means ±SD). Prior to statistical analysis, all parameters were adjusted for gender and age. BMI – body mass index; BW – body weight; MRI – magnetic resonance imaging; MRS – magnetic resonance spectroscopy; SNP – single nucleotide polymorphism. (DOCX) [file pone.0061903.s006.docx]

**Table S3. Association of *FNDC5* SNPs rs16835198, rs3480, rs726344, and rs1746661 with body fat content and body fat distribution**

|  | Genotype | N Overall study group | BMI (kg/m²) | Body fat (%) | Waist circum-ference (cm) | N MRI/MRS subgroup | Total adipose tissue (% BW) | Visceral adipose tissue (% BW) | Intrahepatic lipids (%) |
| --- | --- | --- | --- | --- | --- | --- | --- | --- | --- |
| rs16835198 | GG | 844 | 30.1 ±9.1 | 32.3 ±12.2 | 96 ±19 | 143 | 30.7 ±9.1 | 3.43 ±1.87 | 6.37 ±7.29 |
|  | GT | 892 | 30.1 ±9.4 | 32.8 ±12.2 | 95 ±19 | 177 | 30.0 ±9.1 | 3.25 ±1.66 | 5.06 ±5.12 |
|  | TT | 238 | 30.8 ±9.8 | 33.5 ±11.8 | 96 ±20 | 39 | 32.3 ±9.4 | 3.36 ±1.68 | 7.67 ±7.91 |
|  | - | - | β=0.0051 p=0.6 | β=0.0113 p=0.3 | β=-0.0045 p=0.6 | - | β=0.0155 p=0.5 | β=-0.0095 p=0.8 | β=0.0284 p=0.7 |
| rs3480 | AA | 689 | 30.1 ±9.7 | 32.9 ±11.8 | 95 ±20 | 115 | 30.3 ±9.6 | 3.22 ±1.76 | 5.92 ±6.61 |
|  | AG | 928 | 30.3 ±9.1 | 32.6 ±12.2 | 96 ±19 | 188 | 30.1 ±8.9 | 3.38 ±1.70 | 5.27 ±5.53 |
|  | GG | 355 | 30.0 ±9.3 | 32.3 ±12.4 | 96 ±19 | 54 | 32.4 ±9.0 | 3.41 ±1.89 | 7.49 ±8.30 |
|  | - | - | β=0.0022 p=0.8 | β=0.0038 p=0.7 | β=0.0038 p=0.6 | - | β=0.0282 p=0.2 | β=0.0289 p=0.4 | β=0.0010 p=1.0 |
| rs726344 | GG | 1,590 | 30.2 ±9.4 | 32.6 ±12.0 | 96 ±19 | 280 | 30.3 ±9.0 | 3.34 ±1.81 | 6.05 ±6.47 |
|  | GA | 359 | 30.2 ±9.1 | 32.9 ±12.9 | 96 ±19 | 74 | 31.2 ±9.8 | 3.33 ±1.50 | 4.80 ±5.66 |
|  | AA | 22 | 29.5 ±10.2 | 31.2 ±11.5 | 95 ±23 | 3 | 33.3 ±7.1 | 2.90 ±0.27 | 10.07 ±14.75 |
|  | - | - | β=-0.0020 p=0.9 | β=0.0030 p=0.9 | β=0.0044 p=0.7 | - | β=0.0136 p=0.7 | β=0.0211 p=0.7 | β=-0.1721 p=0.2 |
| rs1746661 | GG | 1,240 | 30.1 ±9.3 | 32.8 ±12.2 | 95 ±19 | 222 | 30.4 ±9.3 | 3.27 ±1.69 | 5.71 ±6.32 |
|  | GT | 627 | 30.4 ±9.4 | 32.4 ±11.9 | 97 ±20 | 117 | 30.2 ±9.2 | 3.44 ±1.78 | 5.52 ±5.93 |
|  | TT | 105 | 30.5 ±8.9 | 33.0 ±12.6 | 97 ±18 | 18 | 35.1 ±7.0 | 3.37 ±2.18 | 9.21 ±9.43 |
|  | - | - | β=0.0121 p=0.3 | β=0.0113 p=0.4 | β=0.0082 p=0.4 | - | β=0.0383 p=0.1 | β=0.0289 p=0.5 | β=0.0563 p=0.6 |

Data are shown as unadjusted raw data (means ±SD). Prior to statistical analysis, all parameters were adjusted for gender and age. BMI – body mass index; BW – body weight; MRI – magnetic resonance imaging; MRS – magnetic resonance spectroscopy; SNP – single nucleotide polymorphism
