# Supplementary material for: Common Genetic Variation in the Human FNDC5 Locus, Encoding the Novel Muscle-Derived ‘Browning’ Factor Irisin, Determines Insulin Sensitivity
Source: PLoS One. 2013 Apr 25;8(4):e61903. doi: 10.1371/journal.pone.0061903 (PMC3636229; doi:10.1371/journal.pone.0061903)
Supplement: Table S4 — Association of FNDC5 SNPs rs16835198, rs3480, rs726344, and rs1746661 with insulin release. Data are shown as unadjusted raw data (means ±SD). Prior to statistical analysis, all parameters were adjusted for gender, age, percentage of body fat, and OGTT-derived insulin sensitivity. AIR – acute insulin response; AUC – area under the curve; C-Pep – C-peptide; Glc – glucose; Ins – insulin; IVGTT – intravenous glucose tolerance test; OGTT – oral glucose tolerance test; SNP – single nucleotide polymorphism. (DOCX) [file pone.0061903.s007.docx]

**Table S4. Association of *FNDC5* SNPs rs16835198, rs3480, rs726344, and rs1746661 with insulin release**

|  | Genotype | N Overall study group | AUC_Ins 0-30_/ AUC_Glc 0-30_ OGTT (*10^-9^) | AUC_C-Pep 0-120_/ AUC_Glc 0-120_ OGTT (*10^-9^) | N IVGTT subgroup | AIR IVGTT (pmol/L) |
| --- | --- | --- | --- | --- | --- | --- |
| rs16835198 | GG | 844 | 46.5 ±36.3 | 324 ±106 | 120 | 955 ±629 |
|  | GT | 892 | 44.6 ±32.7 | 320 ±105 | 150 | 871 ±581 |
|  | TT | 238 | 45.2 ±32.0 | 323 ±108 | 34 | 1,143 ±816 |
|  | - | - | β=-0.0003 p=1.0 | β=-0.0018 p=0.9 | - | β=0.0060 p=0.9 |
| rs3480 | AA | 689 | 45.2 ±32.7 | 322 ±109 | 96 | 985 ±712 |
|  | AG | 928 | 44.5 ±32.6 | 321 ±103 | 159 | 850 ±519 |
|  | GG | 355 | 48.8 ±40.6 | 325 ±106 | 49 | 1,116 ±763 |
| p_add_/p_dom_ | - | - | β=0.0061 p=0.6 | β=-0.0011 p=0.9 | - | β=0.0089 p=0.9 |
| rs726344 | GG | 1,590 | 45.0 ±33.3 | 320 ±107 | 234 | 921 ±635 |
|  | GA | 359 | 47.4 ±38.2 | 327 ±102 | 67 | 970 ±619 |
|  | AA | 22 | 50.0 ±34.7 | 320 ±96 | 3 | 1,217 ±1,013 |
|  | - | - | β=-0.0019 p=0.9 | β=0.0048 p=0.7 | - | β=0.0497 p=0.5 |
| rs1746661 | GG | 1,240 | 44.4 ±31.2 | 321 ±106 | 189 | 937 ±642 |
|  | GT | 627 | 47.4 ±39.2 | 324 ±106 | 97 | 920 ±616 |
|  | TT | 105 | 47.5 ±36.6 | 321 ±107 | 18 | 995 ±673 |
|  | - | - | β=0.0075 p=0.6 | β=-0.0014 p=0.9 | - | β=-0.0060 p=0.9 |

Data are shown as unadjusted raw data (means ±SD). Prior to statistical analysis, all parameters were adjusted for gender, age, percentage of body fat, and OGTT-derived insulin sensitivity. AIR – acute insulin response; AUC – area under the curve; C-Pep – C-peptide; Glc – glucose; Ins – insulin; IVGTT – intravenous glucose tolerance test; OGTT – oral glucose tolerance test; SNP – single nucleotide polymorphism
