# Supplementary material for: Common Genetic Variation in the Human FNDC5 Locus, Encoding the Novel Muscle-Derived ‘Browning’ Factor Irisin, Determines Insulin Sensitivity
Source: PLoS One. 2013 Apr 25;8(4):e61903. doi: 10.1371/journal.pone.0061903 (PMC3636229; doi:10.1371/journal.pone.0061903)
Supplement: Table S5 — Association of FNDC5 SNPs rs16835198, rs3480, rs726344, and rs1746661 with glycaemia and insulin sensitivity (raw data). Data are shown as unadjusted raw data (means ±SD). HOMA-IR – homeostasis model assessment of insulin resistance; ISI – insulin sensitivity index; OGTT – oral glucose tolerance test; SNP – single nucleotide polymorphism. (DOCX) [file pone.0061903.s008.docx]

**Table S5. Association of *FNDC5* SNPs rs16835198, rs3480, rs726344, and rs1746661 with glycaemia and insulin sensitivity (raw data)**

| SNP | Genotype | N Overall study group | Fasting glucose (mmol/L) | Glucose 120 min OGTT (mmol/L) | Fasting insulin (pmol/L) | HOMA-IR (*10^-6^ mol*U*L^-2^) | ISI OGTT (*10^15^ L^2^*mol^-2^) | N Clamp subgroup | ISI Clamp (*10^6^ L*kg^-1^*min^-1^) |
| --- | --- | --- | --- | --- | --- | --- | --- | --- | --- |
| rs16835198 | GG | 844 | 5.14 ±0.57 | 6.34 ±1.67 | 73.7 ±63.5 | 2.90 ±2.69 | 15.0 ±10.5 | 209 | 0.085 ±0.059 |
|  | GT | 892 | 5.14 ±0.55 | 6.36 ±1.61 | 69.3 ±57.4 | 2.71 ±2.42 | 15.2 ±10.2 | 221 | 0.086 ±0.055 |
|  | TT | 238 | 5.15 ±0.51 | 6.40 ±1.66 | 69.3 ±63.4 | 2.75 ±2.89 | 15.5 ±10.8 | 55 | 0.073 ±0.045 |
| rs3480 | AA | 689 | 5.13 ±0.55 | 6.41 ±1.67 | 70.3 ±63.0 | 2.78 ±2.77 | 15.5 ±10.6 | 159 | 0.083 ±0.049 |
|  | AG | 928 | 5.15 ±0.55 | 6.32 ±1.62 | 70.7 ±57.4 | 2.77 ±2.38 | 15.0 ±10.5 | 240 | 0.085 ±0.056 |
|  | GG | 355 | 5.13 ±0.55 | 6.37 ±1.67 | 74.1 ±65.1 | 2.90 ±2.77 | 14.7 ±9.7 | 86 | 0.086 ±0.065 |
| rs726344 | GG | 1,590 | 5.13 ±0.54 | 6.35 ±1.64 | 70.2 ±60.2 | 2.75 ±2.55 | 15.3 ±10.4 | 381 | 0.082 ±0.051 |
|  | GA | 359 | 5.21 ±0.59 | 6.39 ±1.65 | 75.0 ±62.2 | 3.00 ±2.75 | 14.2 ±10.0 | 94 | 0.090 ±0.059 |
|  | AA | 22 | 5.16 ±0.57 | 6.40 ±1.89 | 83.5 ±81.8 | 3.33 ±3.37 | 16.4 ±13.5 | 9 | 0.139 ±0.137 |
| rs1746661 | GG | 1,240 | 5.15 ±0.55 | 6.38 ±1.67 | 70.2 ±60.7 | 2.77 ±2.62 | 15.3 ±10.6 | 304 | 0.088 ±0.060 |
|  | GT | 627 | 5.12 ±0.54 | 6.28 ±1.58 | 71.6 ±59.7 | 2.79 ±2.49 | 14.9 ±10.0 | 151 | 0.077 ±0.047 |
|  | TT | 105 | 5.16 ±0.58 | 6.58 ±1.75 | 79.7 ±68.2 | 3.13 ±2.86 | 14.5 ±9.9 | 30 | 0.080 ±0.044 |

Data are shown as unadjusted raw data (means ±SD). HOMA-IR – homeostasis model assessment of insulin resistance; ISI – insulin sensitivity index; OGTT – oral glucose tolerance test; SNP – single nucleotide polymorphism
